# Culturally adapted depression education and engagement in treatment among Hispanics in primary care: outcomes from a pilot feasibility study

**DOI:** 10.1186/s12875-019-1031-7

**Published:** 2019-10-21

**Authors:** Katherine Sanchez, Michael O. Killian, Brittany H. Eghaneyan, Leopoldo J. Cabassa, Madhukar H. Trivedi

**Affiliations:** 1 School of Social Work, University of Texas at Arlington, 211 South Cooper Street, Arlington, TX 76019 USA; 2 Department of Psychiatry, UT Southwestern Medical Center, Dallas, TX USA; 3 College of Social Work, Florida State University, Tallahassee, FL USA; 4 George Warren Brown School of Social Work, Washington University in St. Louis, St. Louis, MO USA

**Keywords:** Depression, Education, Hispanics, Stigma, Primary care, *Fotonovela*

## Abstract

**Background:**

Low use of anti-depressant medication, poor doctor-patient communication, and persistent stigma are key barriers to the treatment of depression in Hispanics. Common concerns include fears about the addictive and harmful properties of antidepressants, worries about taking too many pills, and the stigma attached to taking medications and seeking mental health treatments. In 2014, the Center for Medicare and Medicaid Services (CMS) funded the *Depression Screening and Education: Options to Reduce Barriers to Treatment (DESEO)* project to implement an education intervention designed to increase disease literacy and dispel myths about depression and its treatment among Hispanic patients thus reducing stigma and increasing treatment engagement.

**Methods:**

The *DESEO* study utilized a one-group pretest-posttest design to assess the effects a culturally-adapted Depression Education Intervention’s (DEI) on depression knowledge, stigma, and engagement in treatment in a sample of 350 Hispanic primary care patients with depression. The DEI utilized a *fotonovela*, a health education tool available in English and Spanish that uses posed photographs, captions, and soap opera narratives to raise awareness about depression and depression treatments.

**Results:**

Participants reported significant decreases in depression symptoms and reported stigma about mental health care. Additionally, participants reported increased knowledge of depression yet greater negative perceptions about antidepressant medication. Finally, 89.5% of participants reported entering some form of treatment at follow-up.

**Conclusions:**

Culturally adapted depression education shows promise in increasing understanding of depression, decreasing stigma, and increasing treatment engagement among Hispanic patients in a community-based health center. Results have implications for practice in addressing common concerns about depression treatments which include fears about the addictive and harmful properties of antidepressants, worries about taking too many pills, and the stigma attached to taking psychotropic medications.

**Trial registration:**

The study was retrospectively registered with www.clinicaltrials.gov: NCT02491034 July 2, 2015.

## Background

The World Health Organization estimates mental disorders collectively account for more than 13% of the global burden of disease from all causes, are the leading cause of disability and associated with the highest rates of unemployment of all disabilities [1]. One in six Americans will experience depression in their lifetime, and recurrence is common, costing an estimated $83–125 billion in the U.S. each year, more than half of which is publicly funded [2, 3]. Virtually all Medicare spending growth in recent years is associated with patients who were treated for five or more conditions, including depression [3].

Individuals with depression are at greater risk for both cardiovascular diseases and type II diabetes [4]. Adequately treating patients with depression and comorbid chronic medical illnesses, like diabetes, can improve morbidity and decrease costs. Safety-net public mental health resources are at capacity, and there remains a significant unmet need [5]. Primary care is well-positioned to deliver services for common mental disorders and, because of insufficient capacity of both primary and specialty care, Federally Qualified Health Centers (FQHCs) play an essential role in promoting access to preventive and primary care among medically underserved populations, including Medicaid, Medicare and the Children’s Health Insurance Program (CHIP) enrollees [6].

Hispanics residing in the United States experience mental health disorders at a rate of 28.1% for men and 30.2% for women [7]. Factors associated with diagnosis of a psychiatric disorder among Hispanic populations include being born in the United States and English proficiency [8]. Hispanics have a higher prevalence of diabetes and comorbid depression which may take significantly longer to treat effectively [9, 10]. Depression relapse rates are high and response to treatment is often slow among Hispanic populations, which often results in the discontinuation of medication [10]. Hispanics experience a considerable burden of disease compared to non-Hispanic whites [10, 11].

Lack of uptake in anti-depressant medication use and stigma, in conjunction with poor communication, are key barriers to depression treatment in Hispanic populations [10, 12, 13]. Common concerns about treatment include fears about the addictive and harmful properties of antidepressants, worries about taking too many pills, and the stigma attached to taking medications [14–16]. Additional barriers include lack of insurance, costs of medications, absence of Spanish-speaking staff, and concerns about immigration status [17, 18].

The Center for Medicare and Medicaid Services (CMS) funded the *Depression Screening and Education: Options to Reduce Barriers to Treatment (DESEO)* project to implement a depression education intervention among Hispanic patients with the goal of reducing stigma and increasing treatment engagement [19]. In this analysis of the primary outcomes from DESEO we sought to examine, does the introduction of a culturally-adapted education intervention after screening positive for depression improve knowledge of the disorder and reduce stigma? Additionally, does the Depression Education Intervention (DEI) lead to subsequent engagement in treatment (any kind) for depression? Our hypothesis was that the provision of culturally-adapted depression education to Hispanic patients in a community-based health center would lead to an increase in engagement in treatment.

## Methods

### Study design and setting

The DESEO study utilized a one-group pretest-posttest design to assess a culturally-adapted DEI effects on depression knowledge, stigma, and engagement in treatment within a sample of Hispanic primary care patients diagnosed with depression [19]. The study took place in an FQHC in a large metropolitan area in Texas between February 2015 and October 2016. The community clinic provides comprehensive primary care to low-income, primarily Hispanic populations. Behavioral health services are provided by a Licensed Clinical Social Worker (LCSW) and include brief counseling using evidence-based interventions such cognitive behavioral therapy and behavioral activation. The study was reviewed and approved by the Institutional Review Board of the University of Texas at Arlington.

### Sample

From February 2015 through October 2016, all adult primary care patients were universally screened for depression using the Patient Health Questionnaire-9 (PHQ-9) [20], during annual or new/non-acute visits. Patients who were 18 years or older, self-identified as Hispanic, met diagnostic criteria for depression, and were not already receiving treatment were invited to participate in the study [19]. The final study sample included 350 Hispanic participants. For further detail on sample acquisition see Lopez, et al. [21]

### Intervention

The DEI offered to all participants utilized a comic-book style pamphlet titled “Secret Feelings” developed by Cabassa, Molina, and Baron [22]. The *fotonovela* was presented in both English and Spanish at a 4th grade reading level and uses an entertainment-education approach to portray the story of a Latina woman experiencing depression as she describes her symptoms, presents common fears and misconceptions (addiction, sexual dysfunction), and displays both informal and formal help-seeking behaviors (a friend and a trusted pharmacist) as she discusses her mental health concerns with her family and engages in formal depression treatment [21, 23]. The *fotonovela* has been tested via two randomized trials in clinical and non-clinical settings demonstrating its effectiveness to decrease stigma toward mental health care and increase knowledge of depression [23–25].

The clinic’s bilingual LCSW served as the Depression Educator (DE) and read the *fotonovela* with the participants, using it to promote discussion centered around the participants’ own experiences with depression, treatment options, and stigmas and fears concerning potential treatments. The DEI was delivered in English or Spanish and in the presence of family members or loved ones, if desired. At the conclusion of the discussion, the DE invited participants to participate in the decision-making process for their own depression treatment and offered treatment options including behavioral activation strategies (walking, gardening, visiting with friends), counseling with the DE, and/or antidepressant medication. The DE could facilitate a warm hand off back to the provider if the patient expressed an interest in antidepressants and the primary care provider could prescribe the medication.

### Procedures

Universal screening for depression was implemented for all adult patients during visits with their primary care providers using the 9-item Patient Health Questionnaire (PHQ-9) [20]. The DE administered four additional baseline measures including the Depression Knowledge Measure (DKM) [25] and three stigma measures (see below). All informed consent documents and measures were offered in English or Spanish based on patient preference. Detailed procedures for the study can be found in Sanchez, et al. [19]

**Fig. 1:**
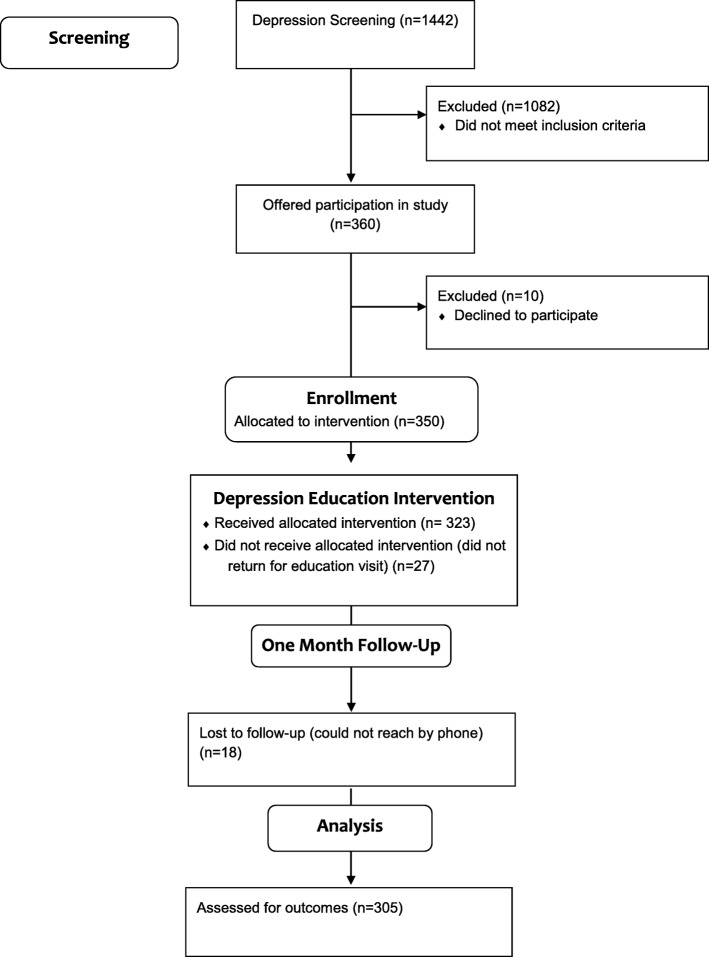
DESEO study flow diagram

When the participants returned for their DEI session, the DE administered the PHQ-9 prior to the education intervention, and the DKM and three stigma measures afterwards. One month after the DEI session, participants were contacted by the DE or a student research assistant to complete the final measures by phone including the PHQ-9, DKM, three stigma measures, and to assess whether they had engaged in treatment. See Fig. 1 for study flow diagram.

### Measures

#### Depression

Depression severity was measured using the PHQ-9, a self-report measure that assesses the frequency of depression symptoms within the last two weeks using each of the of the nine Diagnostic and Statistical Manual of Mental Disorders (DSM-IV) [26] criteria for depression. Total scores range from 0 to 27 with a PHQ-9 score > 10 are considered clinically significant depressive symptoms. The PHQ-9 has demonstrated to be a reliable and valid measure of depression severity in racially and ethnically diverse primary care samples [27].

#### Depression knowledge

The DKM was developed by Unger et al. [25] to evaluate the effect of the *fotonovela*, *Secret Feelings*, on depression knowledge. The measure has a total of 17 items including symptom recognition of five DSM depression symptoms (sleeping too little, eating too much, feeling agitated, feeling guilty, and loss of interest) and five non-depressive symptoms (hearing voices, being full of energy, being violent, having hallucinations, and feeling confident). One point was allocated for each correct response, with total scores ranging from 0 (all incorrect) to 17 (all correct).

#### Stigma

Three measures developed by Interian et al. [28] were used to assess depression stigma. The Stigma Concerns about Mental Health Care (SCMHC) scale is a 3-item scale (possible scores ranging from 0 to 3) used to measure stigma towards depression treatment. The SCMHC has demonstrated internal consistency with a Cronbach’s alpha of .84.

The Latino Scale for Antidepressant Stigma (LSAS) includes seven items measuring stigma towards the use of antidepressant medications. Possible scores for the scale range from 0 to 14, with higher scores indicating greater stigma. The LSAS has demonstrated internal consistency with a Cronbach’s alpha of .80.

Finally, the Social Distance (SD) scale was used to measure social distance desirability from someone with depression or history of depression treatment [29, 30]. The measure has six items with lower scores indicating greater desired social distance (i.e., greater stigma). The SD has demonstrated internal consistency with a Cronbach’s alpha of .75.

#### Treatment engagement

Participants self-reported whether they had engaged in treatment for their depression at the one-month follow-up phone call by responding yes/no to the question “Since meeting with the DE and discussing the *fotonovela*, have you participated in some type of treatment for your depression?” If the patient endorsed “yes” they were engaged in treatment, additional response choices were collected and included 1) taking medication only, 2) receiving counseling only, 3) taking medication and receiving counseling, and 4) other behavioral interventions. “Taking medication” was defined as currently taking medication prescribed to treat depression. “Receiving counseling” was defined as attending at least one counseling session with the DE or other mental health professional after the DEI session. “Other behavioral interventions” included behavioral activation (BA) activities that the DE and the participant set as goals for the treatment of their depression and responses were collected qualitatively.

### Statistical analyses

Testing for differences between the group completing the protocol and those participants dropping out of the study (attritioners) used various bivariate statistical tests (Table 1). Testing for changes in participant reported scores over time used repeated-measures analysis of variance (RMANOVA). Multinomial logistic regression was used to test the association between the self-report measures from follow-up and the likelihood of the treatment engagement categories (i.e., no treatment as the reference category, counseling only or other behavioral interventions, and medication or medication with counseling). Effect sizes were calculated and included partial *ƞ*^2^ and Nagelkerke r.^2^ All tests assumed a significance value of .05 and were completed using SPSS 25.0 software (IBM, 2017).

**Table 1:** Descriptive statistics of sample

	Total Sample (*n* = 350)	Completers (*n* = 305)	Attritioners (*n* = 45)	Test	*p*-value
Age, *M (SD)*	38.81 (10.60)	39.00 (10.00)	37.53 (14.08)	t = 0.676	.502
Gender, female, *n (%)*	327 (93.4%)	285 (93.4%)	42 (93.3%)	Fisher Exact	.999
Spanish Speaking, yes, *n (%)*	332 (95.1%)	288 (94.7%)	44 (97.8%)	Fisher Exact	.708
Marital Status, *n (%)*				*X*^2^=4.289	.368
*Married/cohabitating*	247 (70.6%)	217 (71.1%)	30 (66.7%)		
*Never married*	39 (11.1%)	36 (11.8%)	3 (6.7%)		
*Widowed*	10 (2.9%)	8 (2.6%)	2 (4.4%)		
*Divorced*	29 (8.3%)	25 (8.2%)	4 (8.9%)		
*Other*	25 (7.1%)	19 (6.2%)	6 (13.3%)		
Education Level, *n (%)*				*X*^2^=2.014	.847
*8th grade or less*	126 (36.8%)	111 (36.4%)	15 (40.5%)		
*Some high school*	92 (26.9%)	83 (27.2%)	9 (24.3%)		
*High school or GED*	75 (21.9%)	67 (22.0%)	8 (21.6%)		
*Vocational or trade school*	9 (2.6%)	7 (2.3%)	2 (5.4%)		
*Some college*	30 (8.8%)	28 (9.2%)	2 (5.4%)		
*College degree*	10 (2.9%)	9 (3.0%)	1 (2.7%)		
Patient Health Questionnaire, *M (SD)*	17.55 (3.89)	17.53 (3.70)	17.69 (4.99)	t = 0.206	.838
*No depression, n (%)*	0 (0%)	0 (0%)	0 (0%)	*X*^2^=2.502	.475
*Mild depression, n (%)*	7 (2.0%)	5 (1.6%)	2 (4.4%)		
*Moderate depression, n (%)*	66 (18.9%)	57 (18.7%)	9 (20.0%)		
*Moderately severe depression, n (%)*	167 (47.7%)	149 (48.9%)	18 (40.0%)		
*Severe depression, n (%)*	110 (31.4%)	94 (30.8%)	16 (35.6%)		
Depression Knowledge Measure, *M (SD)*	10.76 (2.15)	10.81 (2.15)	10.44 (2.15)	t = 1.053	.293
Stigma Concerns About Mental Health Care, *M (SD)*	.44 (.85)	.43 (.84)	.47 (.89)	t = 0.239	.811
Latino Scale for Antidepressant Stigma, *M (SD)*	6.13 (3.45)	6.12 (3.42)	6.18 (3.72)	t = 0.107	.915
Social Distance, *M (SD)*	9.00 (3.07)	9.05 (2.99)	8.64 (3.61	t = 0.831	.407
Treatment Outcome and Engagement, *n (%)*					
*Medication or counselling with medication*	106 (34.8%)	–	–		
*Counseling or other behavioral intervention*	167 (54.8%)	–	–		
*No treatment at follow-up*	32 (34.8%)	–	–		

## Results

### Sample characteristics

At baseline, nearly half of the sample participants (*N* = 350) reported moderately severe depression scores (*n* = 167, 47.7%) with only 2 % of the sample reporting less than moderate depression. Severe depression scores were reported by 31.4% of the sample (*n* = 110), but there were no reported adverse events or psychiatric emergencies related to suicidal ideation or self-harming. The sample was nearly all women (*n* = 327, 93.4%) with a mean age of 38.81 years (SD = 10.60, range 18 to 82 years). The sample was entirely Hispanic and their country of origin unknown, though 88% of Hispanics in Texas are of Mexican descent [31]. See Table 1. No significant differences were found between those completing the protocol (*n* = 305, 87.1%) and those who dropped out (*n* = 45, 12.9%) based on their demographic characteristics or baseline measurement in the study (Table 1).

### Self-report measures: changes over time

The descriptive statistics for the primary outcomes related to changes in stigma and depression knowledge are provided in Table 2 for those participants who completed the protocol (*n* = 305). Stigma Concerns About Mental Health Care (F [1.907, 574.076] = 21.914, *p* < .001, partial *ƞ*^2^=.068) significantly decreased over time. Similarly, social distance scores significantly increased over time (F [1.823, 548.637] = 50.288, *p* < .001, partial *ƞ*2 = .143) indicating less desire for social distance from those with mental health disorders and less stigma, as did depression knowledge (F [1.886, 563.768] = 807.305, *p* < .001, partial *ƞ*2 = .730). Latino Scale for Antidepressant Stigma scores significantly increased over time (F [2, 588] = 14.633, *p* < .001, partial *ƞ*^2^=.047) indicating greater concerns about others’ negative perception of the use of psychiatric medication (Table 2).

**Table 2:** Changes in self-reported measures over time (*n* = 305)

	Baseline	2nd Session	One month follow-up	F	Partial *ƞ*^2^
Depression Knowledge Measure, *M (SD)*	10.81 (2.15)	15.71 (1.70)	14.99 (1.82)	807.305***	.730
Stigma Concerns About Mental Health Care, *M (SD)*	.43 (.84)	.27 (.67)	.10 (.43)	21.914***	.068
Latino Scale for Antidepressant Stigma, *M (SD)*	6.12 (3.42)	6.56 (3.46)	7.19 (2.62)	14.633***	.047
Social Distance, *M (SD)*	9.05 (2.99)	9.92 (2.61)	10.52 (2.24)	50.288***	.143
Patient Health Questionnaire, *M (SD)*	17.53 (3.70)	14.77 (5.11)	9.47 (6.04)	319.328***	.516
*No depression, n (%)*	0 (0%)	10 (3.3%)	77 (25.2%)	–	
*Mild depression, n (%)*	5 (1.6%)	44 (14.4%)	89 (29.2%)	–	
*Moderate depression, n (%)*	57 (18.7%)	82 (26.9%)	69 (22.6%)	–	
*Moderately severe depression, n (%)*	149 (48.9%)	116 (38.0%)	54 (17.7%)	–	
*Severe depression, n (%)*	94 (30.8%)	50 (16.4%)	16 (5.2%)	–	

*** *p* < .001

Analysis of depression scores indicated participants reported significantly lower scores on the PHQ-9 (F [1.876, 562.718] = 319.328, *p* < .001, partial *ƞ*^2^=.516) at each time point. Effect sizes (partial *ƞ*^2^) indicated moderate changes in scores. At baseline, 79.7% (*n* = 243) of the sample reported moderately severe or severe depression, and less than a quarter of the sample reported similar depression scores at follow-up (*n* = 70, 22.9%). A majority of the sample reported no or mild depression at follow-up (*n* = 166, 54.4%) when only five participants reported these levels at baseline (1.6%) (Table 2).

### Treatment engagement

A majority of participants reported entering some form of treatment at follow-up (*n* = 273 of 305, 89.5%). Almost 20% of participants (*n* = 58) reported attending some form of counseling without medication while a third reported either being prescribed medication or a combination of medication and counseling (*n* = 106, 34.8%). Another third of the sample (*n* = 109, 35.7%) reported engaging in BA activities which included dietary changes, exercise, meditation, yoga, and spending more time socializing with loved ones. Only about 10% of the sample reported not entering treatment following the intervention (*n* = 32, 10.5%).

Self-reported treatment engagement at 30-day follow-up was coded as taking medication or counseling with medication, counseling only or other behavioral interventions, or reporting not engaged in any treatment (Table 3). At 30-day follow-up after the education intervention, PHQ-9 (*X*^2^=34.98, df = 2, *p* < .001, Nagelkerke r^2^ = .128) and DKM scores (*X*^2^=11.23, df = 2, *p* = .004, Nagelkerke r^2^ = .043) significantly predicted likelihood of treatment engagement. Specifically, for every one-point increase in depression knowledge scores, participants were 28.8% more likely to engage in counseling (*β*=.253, Wald t = 7.589, df = 1, *p* = .006, OR = 1.288) and 40.9% more likely to report taking medication (*β*=.343, Wald t = 11.235, df = 1, *p* = .001, OR = 1.409). A one-point increase in DKM score represents correct identification of a depression symptom or knowledge of the disorder, and greater overall knowledge. Participants with higher depression scores at 30-day follow-up were more likely to report engaging in medication treatment than those participants reporting no treatment engagement at follow-up (*β*=.102, Wald t = 8.122, df = 1, *p* = .004, OR = 1.108).

**Table 3:** Bivariate and multivariate multinomial regression modeling of treatment engagement predicted by 30-day follow up scores (*n* = 305)

	Model *X*^2^	Nagelkerke r^2^	Counseling + Behavioral Intervention^a^					Medication or Meds+Counseling^a^				
			β	Wald t	OR	95%CI lower	95%CI upper	β	Wald t	OR	95%CI lower	95%CI upper
Bivariate^a^												
PHQ-9	34.98***	.128	−.025	.525	.975	.912	1.043	.102	8.122**	1.108	1.032	1.188
DKM	11.23**	.043	.253	7.589**	1.288	1.076	1.542	.343	11.235**	1.409	1.153	1.723
SCMHC	2.684	.010	−.386	1.375	.680	.356	1.296	−.678	2.717^+^	.508	.227	1.137
LSAS	.677	.003	.015	.037	1.015	.874	1.178	−.025	.098	.975	.835	1.139
SD	.367	.001	.020	.058	1.020	.869	1.197	.047	.290	1.048	.883	1.243
Multivariate^ab^	49.305***	.177										
PHQ-9			.252	7.210**	1.286	1.070	1.546	.403	14.066***	1.497	1.212	1.848
DKM			−.011	.093	.989	.924	1.059	.123	10.964**	1.131	1.051	1.216

^+^ *p* < .10

** *p* < .01

*** *p* < .001

^a^Reference category is no treatment

^b^Both PHQ-9 and DKM scores entered into same model in a multivariate model

After controlling for depressions scores at the 30-day follow-up, depression knowledge still predicted treatment engagement in a multivariate model. Participants were 28.6% more likely to engage in counseling (*β*=.252, Wald t = 7.210, df = 1, *p* = .007, OR = 1.286) and 49.7% more likely to report taking medication (*β*=.403, Wald t = 14.066, df = 1, *p* < .001, OR = 1.497) compared to those who reported no treatment engagement (Table 3).

## Discussion

Management of depression among Hispanics in community-based clinics is confounded by issues of disease literacy, cultural treatment preferences and financial barriers to care [21, 32, 33]. The culturally-adapted depression education *fotonovela* delivered by an LCSW in this primary care study appeared to increase knowledge of the disorder, reduced some stigma indicators, particularly related to mental health care use and desire for social distance from people with mental disorders, and lead to significant engagement in treatment for depression. The unexpected finding related to increased stigma toward antidepressant use may reflect the knowledge gained about sexual dysfunction and other side effects, and awareness of stigmatizing attitudes toward medication in general. These findings warrant further exploration, perhaps qualitatively, as side effects pose considerable challenges to treatment, often lead to the use of subtherapeutic doses, poor treatment adherence and quality of life [34].

While two previous randomized trials of “Secret Feelings” have produced improvements in depression knowledge and reductions in stigma indicators particularly stigma toward mental health care in nonclinical, adult education classes and in a community center among Hispanic adult women at risk for depression but not receiving mental health care [23–25], this is the first application of the depression *fotonovela* in primary care with patients diagnosed with clinical depression. The *fotonovela* concept has been widely recognized as an effective public health tool among Hispanics in the US and Latin America [35, 36] and recommended as a strategy for encouraging participation of Hispanics in research [37] however, its efficacy as a patient education intervention to improve depression literacy and treatment engagement in clinical settings has not been tested.

Shared decision making requires providers assess the patient’s interest in initiating treatment, provide information on the risks and benefits of specific treatments in an understandable format, and discuss with patients their choices [38]. For patients struggling with stigma and fears about psychotropic medications, communication can be improved by adopting patient-centered approaches, which are essential to improve the quality of treatment and may improve outcomes [37, 39, 40].

Patients play a critical role in self-management of their illnesses and educating them to understand the behavioral and psychosocial elements of managing their disease is the first step to help improve opportunities for optimal health [41]. Our findings indicated a significant relationship between knowledge gained and subsequent engagement in counseling, adding to the evidence which suggests that decision aids, such as culturally-appropriate patient education can improve people’s knowledge regarding options, and reduce their decisional conflict related to feeling uninformed and unclear about their personal values [42].

This study has some limitations. The funding opportunity from the CMS *Hispanic Health Services Research Grant Program* specified that funded projects be for educational intervention studies, which would inform populations-at-risk and that the intervention reach a minimum of 350 participants. This requirement made a comparison group from the same site impossible and introduced the limitations of a one-group, pretest-posttest design [19] There was also only one LCSW available at the clinic to train as the DE, so the effects of the clinician cannot be separated from the education intervention itself. As with all research studies, and primary care in general, a few participants were lost between the enrollment and the education visit (*n* = 27) and at one-month follow-up (*n* = 18), however these losses were attributed to typical life circumstances (phone disconnected, patient relocated) and the overall study retention rate was quite high (87%). Additionally, the outcome measure of engagement in depression treatment at one month follow up was a self-report item collected via phone, which introduces inaccuracy and potential response bias.

## Conclusions

Interventions in primary care that aim to educate patients in culturally meaningful ways may facilitate early identification of barriers and result in better engagement in treatment, especially for disparity populations. Culturally appropriate tools which address person-level barriers can lead to active engagement of the patient and family members in shared decision-making regarding treatment and offer solutions for the patient to adhere to treatment, share information about response and side effects, and facilitate decision support [38, 43, 44]. Findings from the current study provided sufficient pilot data to lead to a National Institutes of Health funded rigorous evaluation of the *fotonovela* intervention in a randomized control trial, an important next step [45]. More research is needed on implementation of evidence-based guidelines at the provider and systems levels to improve treatment options and patient outcomes in the Hispanic community.

## Data Availability

The datasets used and/or analyzed during the current study are available from the corresponding author on reasonable request.

## Abbreviations

BA: Behavioral activation
CMS: Center for Medicare and Medicaid Services
DESEO: Depression Screening and Education: Options to Reduce Barriers to Treatment
DKM: Depression knowledge measure
FQHC: Federally qualified health center
GED: General Education Diploma
LCSW: Licensed Clinical Social Worker
LSAS: Latino Scale for Antidepressant Stigma
PHQ-9: 9-Item Patient Health Questionnaire
PI: Principal Investigator
SCMHC: Stigma Concerns about Mental Health Care
SD: Social Distance scale
